# Improved image quality and diagnostic potential using ultra-high-resolution computed tomography of the lung with small scan FOV: A prospective study

**DOI:** 10.1371/journal.pone.0172688

**Published:** 2017-02-23

**Authors:** Huiyuan Zhu, Lian Zhang, Yali Wang, Preeti Hamal, Xiaofang You, Haixia Mao, Fei Li, Xiwen Sun

**Affiliations:** 1 Department of Radiology, Pulmonary Hospital Affiliated to Tongji University, Shanghai, China; 2 Department of Radiology, Jiading Hospital of Traditional Chinese Medicine, Shanghai, China; Chongqing University, CHINA

## Abstract

The aim of this study was to assess whether CT imaging using an ultra-high-resolution CT (UHRCT) scan with a small scan field of view (FOV) provides higher image quality and helps to reduce the follow-up period compared with a conventional high-resolution CT (CHRCT) scan. We identified patients with at least one pulmonary nodule at our hospital from July 2015 to November 2015. CHRCT and UHRCT scans were conducted in all enrolled patients. Three experienced radiologists evaluated the image quality using a 5-point score and made diagnoses. The paired images were displayed side by side in a random manner and annotations of scan information were removed. The following parameters including image quality, diagnostic confidence of radiologists, follow-up recommendations and diagnostic accuracy were assessed. A total of 52 patients (62 nodules) were included in this study. UHRCT scan provides a better image quality regarding the margin of nodules and solid internal component compared to that of CHRCT (P < 0.05). Readers have higher diagnostic confidence based on the UHRCT images than of CHRCT images (P<0.05). The follow-up recommendations were significantly different between UHRCT and CHRCT images (P<0.05). Compared with the surgical pathological findings, UHRCT had a relative higher diagnostic accuracy than CHRCT (P > 0.05). These findings suggest that the UHRCT prototype scanner provides a better image quality of subsolid nodules compared to CHRCT and contributes significantly to reduce the patients' follow-up period.

## Introduction

With the development of high-resolution CT (HRCT) technology and the widespread use of low-dose CT in lung cancer screening, the detection rate of pulmonary nodules has significantly increased. However, the malignancy of identified nodules is becoming an issue of increasing concern [1–4]. Patients may require a repeated follow-up CT examination if the nodules cannot be clearly diagnosed. The US National Comprehensive Cancer Network (NCCN) guidelines [5] recommend the reexaminations for patients with pulmonary nodule less than 1 cm in size manifesting as ground-glass opacity, ground-glass nodule, and/or nonsolid nodule after 6–12 months. If it is stable, annual follow-up surveillance using CT examination are recommended for at least two years; if it enlarges or appears to have solid components, then surgical intervention is required. The Fleischner Society guidelines recommend annual follow-up surveillance via CT examination at least three years for subsolid pulmonary nodules [6]. However, the long-term follow-up will increase the psychological burden of patients and the risk of high cumulative radiation doses due to excessive scanning. These issues have increased the need for a better CT scanning technique and improved diagnostic capabilities of pulmonary nodules. Previous studies suggest that scanning and reconstruction parameters may affect the spatial resolution of nodule [7, 8], such as section thickness and field of view (FOV). Recently, Ryutaro Kakinuma et al [9] retrospectively compared the image quality of the ultra-high-resolution (UHRCT) with conventional HRCT (CHRCT) and found that UHRCT had better image quality and display details. Sheshadri et al. [10] reported that reducing the FOV could significantly improve image spatial resolution. In this study, we introduced a new UHRCT method. Compared with CHRCT, this method has the following characteristics: 1) the matrix of the scanner is 1024*1024; 2) scanning field of view is 100 mm or less; 3) slice thickness is 1mm or less. This study will further prospectively investigate the advantages of UHRCT scan for the diagnosis of small pulmonary nodules. We hypothesized that it will significantly improve the image quality and help to reduce the follow-up period compared with a conventional high-resolution CHRCT scan.

## Materials and methods

### Patients

The prospective study was approved by the ethical committee of Pulmonary Hospital Affiliated to Tongji University. All the patients provided their written informed consent to participate in this study. In this study, we identified patients with at least one pulmonary nodule who came to our hospital from July 1^st^, 2015 to November 30^th^, 2015. Inclusion criteria was as follows: 1) patients with pulmonary nodule(s) detected by screening, 2) patients had no history of cancer. 3) Both CHRCT and UHRCT scans were performed for all enrolled patients. Three experienced radiologists with 10-year experience in thoracic imaging diagnosis were asked to evaluate the image quality and provide diagnoses. We then compared the advantages of the UHRCT scan over the CHRCT, regarding the diagnosis of lung nodules.

### Protocol for UHRCT and CHRCT scanning

Two CT scanners were applied in this study: 1) 128-row (Brilliance 128, Philips, Holland); and 2) 16-row (uCT 510, United Imaging, China). Two sets of images for each patient were obtained from the same CT scanner. Patients had never been scanned using different CT scanners in this study.

One of the utilizised UHRCT prototype is a 128-row detector CT scanner (Brilliance 128, Philips, Holland). There are three types of matrix size: 512×512, 768×768, 1024×1024. The beam collimation is 0.625 mm×128. The focus size of X-ray tube is 0.6×0.7 mm. The prototype has a maximum output of 175KVA, maximum scanning FOV of 500 mm, and a gantry rotation speed of 0.27s per rotation. Another prototype is a 16-row detector CT scanner (uCT510, United Imaging, China). There are two types of matrix size: 512×512, 1024×1024. The beam collimation is 1.2 mm×16. The focus size of X-ray tube is 0.5×1.0 mm. The prototype has a maximum output of 50 KV, maximum scanning FOV of 500 mm, and a gantry rotation speed of 0.5s per rotation.

The scanning procedures are as follows: 1) patient was placed in dorsal position on the bed; 2) a scanogram was obtained; 3) a CHRCT was performed with a coverage from the apex to the bottom of the lung. The scan parameters for the Philips were 120 KV, 100 mAs, a beam collimation of 0.625 mm × 128, the volumetric CT dose index (CTDI_vol_) of 6.8 mGy, 350-mm field of view (FOV), 0.5-s rotation time, 0.804-mm helical pitch, and a matrix of 512 x 512. For the United Imaging, the parameters were 120 KV, 100 mAs, a beam collimation of 1.2 mm × 16, CTDI_vol_ of 6.8 mGy, 350-mm FOV, 0.8-s rotation time, 1.062-mm helical pitch, and a matrix of 512 × 512. All CHRCT images were reconstructed using 1.0 mm-thick slices on Philips Extended Brilliance Workspace v3.5 (Philips Medical Systems); 4) a UHRCT was performed with a nodule-centered coverage of 50 mm. The scan parameters for the Philips were: 120 KV, 200 mAs, a beam collimation of 0.625 mm × 128, CTDI_vol_ of 13.9 mGy, 100-mm scan FOV, 0.5-s rotation time, and 0.578-mm helical pitch, and a matrix of 1024 × 1024. For United Imaging, the parameters were: 120 KV, 200 mAs, a beam collimation of 1.2 mm × 16, CTDI_vol_ of 14.7 mGy, 100-mm scan FOV, 0.8-s rotation time, 0.937-mm helical pitch, and a matrix 1024 × 1024. All UHRCT images were reconstructed using 1.0 mm-thick slices on Philips Extended Brilliance Workspace v3.5. The lung window settings for both CHRCT and UHRCT were a width of 1200 HU and a center of -450HU.

Patients were required to hold their breath during the examination, and intravenous contrast agent was not used. Since both the CHRCT and UHRCT were obtained in the same scanning program from the same CT scanner, the median interval was 0 days.

### Image analysis

The images were independently evaluated by three experienced radiologists with 10-year experience in thoracic imaging diagnosis. The anonymous UHRCT and CHRCT images of the same nodule from the same CT scanner were shown side by side in a random manner and were sorted by the author after the reading. When they viewed the CT images, the readers were asked to make a diagnosis, evaluate the quality of CT findings and assign scores using a 5-point score as follows:

1. Each CT finding, including the lobulation sign, spiculation, pleural indentation, bubble sign, margin of nodule, solid component was subjectively evaluated and graded using a 5-point score according to a previous report [9]: ‘1’ indicated worst image quality which means no detectable findings; ‘2’ indicated poor image quality that findings can be detected but the margin or internal characteristics are difficult to evaluate; ‘3’ indicated fair image quality in which partially indistinct findings can be detected and the margin or internal characteristics can be evaluated; ‘4’indicated good image quality in which some indistinct findings can be detected and the margin or internal characteristics can be evaluated; and ‘5’ indicated excellent image quality in which findings are extremely clear and easy to detect, and the margin or internal characteristics can be evaluated.
2. Diagnostic confidence: ‘1’ to ‘5’ indicated increasing levels of confidence. ‘1’indicated no confidence; and ‘5’indicated full confidence.
3. Diagnosis: benign; atypical adenomatous hyperplasia (AAH); adenocarcinoma in situ (AIS); minimally invasive adenocarcinoma (MIA); and invasive adenocarcinoma (IAC). Imaging diagnoses were ultimately verified by pathology. Specimens were regularly fixed with 4% neutral formaldehyde, embedded in paraffin, and stained with hematoxylin-eosin (HE).
4. Follow-up recommendations: no necessary for follow up; surgery; or follow-up.

### CT of representative cases

To display the nodule morphology on UHRCT and CHRCT, we performed multi-planar reconstruction (MPR) using a dedicated workstation (Philips Extended Brilliance Workspace v3.5; Philips Medical Systems)

### Statistical analysis

Data analysis was performed using the SPSS17.0 software package (SPSS Inc. Chicago, IL, United States). The diagnosis results, treatment recommendatinos, and diagnosis accuracy were determined using McNemar tests. The Wilcoxon signed-rank test was used to evaluate the image quality scores of the findings and confidence of readers. A P value of less than 0.05 was considered statistically significant.

The inter-observer agreement for the 5-point score and diagnosis described above was evaluated by κ statistics. The κ values were regarded as follows: 0.00–0.20 was poor; 0.21–0.40 was fair; 0.41–0.60 was moderate; 0.61–0.80 was good; and 0.81–1.00 was excellent. In this study, a higher value of κ indicates a higher agreement among the three observers.

## Results

As a result, 52 patients (62 nodules) were included the final analysis, of which 19 were males and 33 were females. The mean age was 46.13 ± 10.42 (range: 28–70 years). Of the 62 nodules performed using UHRCT, 44 nodules were scanned by United Imaging, and the remaining 18 were scanned by the Philips. The mean size of the 62 nodules was 6.18 ± 0.22 mm (ranged from 2.60 to 10.00 mm). Among the 62 nodules, 22 nodules were treated with surgery, including one nodule of benign nodule, eight nodules of AAH, nine nodules of AIS, three nodules of MIA, and one nodule of IAC. Moreover, 17 nodules were considered to be malignant but were not resected, and the remaining 23 nodules were considered as benign lesions or required follow-up.

### Assessment of image quality

Scores for two sets of CT images are shown in Table 1. Since the number of cases with lobulation sign, spiculation, pleural indentation or bubble sign is very small, they were not included in the statistical test. The image quality of the UHRCT findings has significantly higher score regarding the margin of the nodule and solid internal component than that of the CHRCT (all P < 0.05).

**Table 1:** Comparison of scores for the two sets of CT images.

	Lobulation sign	Spiculation	Pleural indentation	Bubble sign	Margin of nodule	Solid component
CHRCT	3.50	2.50	3.14	3.30	3.22±0.42	2.86±0.46
UHRCT	3.86	3.00	4.08	4.00	3.92±0.48	3.29±0.53
P value	-	-	-	-	<0.05	<0.05

CHRCT, conventional high-resolution CT; UHRCT, ultra-high-resolution CT.

### Diagnosis and follow-up recommendations

**Fig 1:**
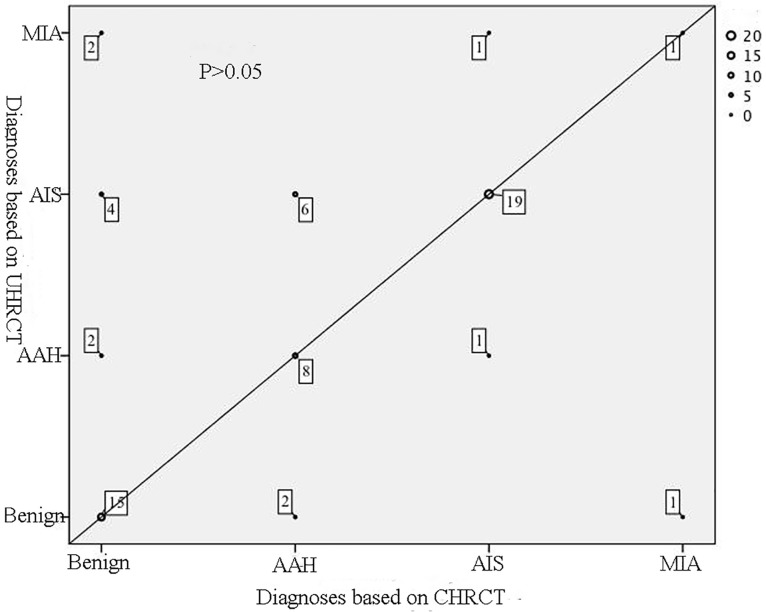
The scatterplot of diagnoses for the two sets of images of each case. Spots on the solid line represents that the diagnoses for the two sets of images are the same. The more spots overlap, the bigger the size of spot. The counts of overlapped spots and the P value are marked.

**Fig 2:**
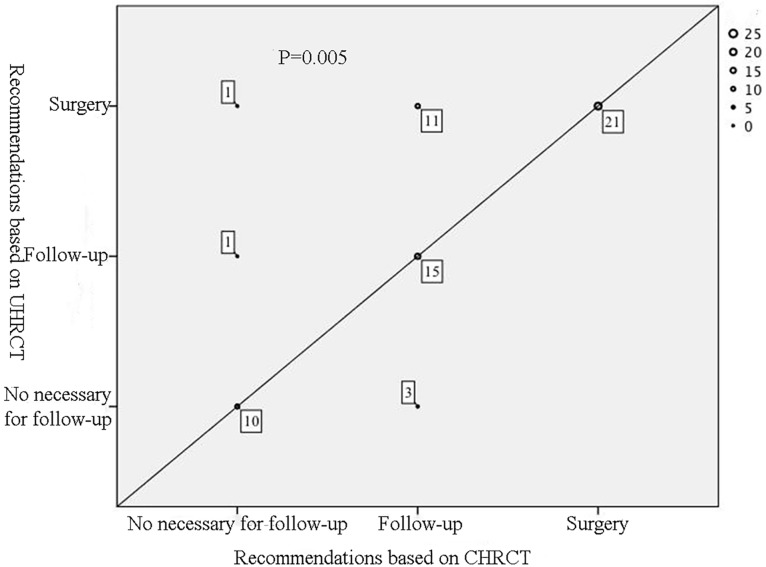
The scatterplot of follow-up recommendations for the two sets of images of each case. Spots on the solid line represents that the follow-up recommendations for the two sets of images are the same. The more spots overlap, the bigger the size of spot. The counts of overlapped spots and the P value are marked.

The results of diagnosis and follow-up recommendations are shown in Figs 1 and 2. There is no statistical significant difference regarding the diagnosis between the UHRCT and CHRCT images (P>0.05). With regard to the follow-up recommendations, three readers gave statistically different results (P<0.05) between the two sets of images. As shown in Fig 2, 14 cases recommending for follow-up based on CHRCT scans were recommended for surgery or no follow-up after UHRCT scans. A total of 12 cases were recommended for surgery after UHRCT scans, only 5 nodules were resected actually including 4 AIS and 1 AAH.

### Diagnostic confidence

**Fig 3:**
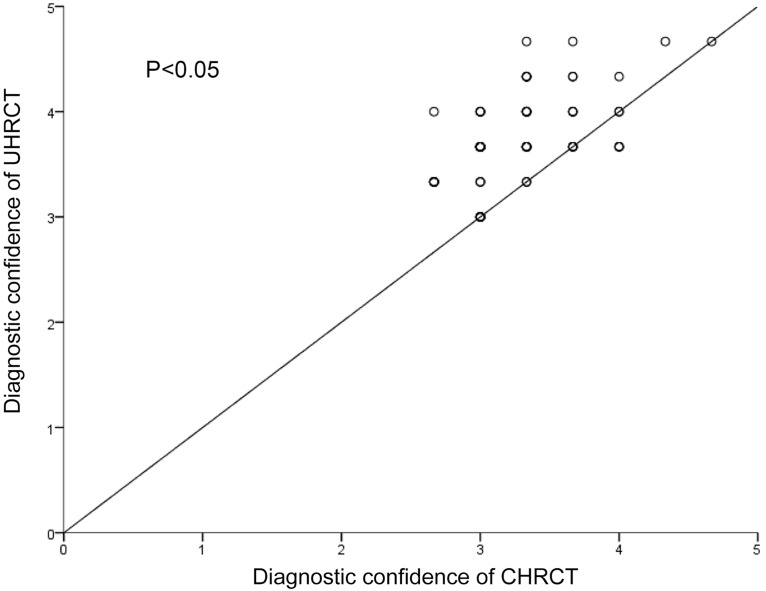
Scatterplot of the mean diagnostic confidence for each nodule. The solid line represents a hypothetical line indicating that the confidence of ultra high-resolution computed tomography (UHRCT) images is equivalent to that of conventional high-resolution computed tomography (CHRCT) images.

A scatterplot of the mean diagnostic confidence for each nodule is shown in Fig 3. As shown in the figure, plots of most nodules are above the line, showing that the diagnostic confidence of UHRCT images is higher than that of CHRCT (P < 0.05).

### Diagnostic accuracy

The three radiologists provided definite diagnoses after discussing (benign diagnoses, including AAH, granuloma, tuberculoma; and malignant diagnoses, including AIS, MIA, IAC). Compared with the surgical pathological findings, UHRCT (14/22) had a higher diagnostic accuracy than that of CHRCT (12/22), although the difference was not statistically significant (P > 0.05).

### Inter-observer agreement

The inter-observer agreement is shown in Table 2. When the three readers were compared in pairs of two readers, three combinations of paired readers were possible. The maximum k value was 1, the minimal k value was -0.02, and the median k value was 0.37.

**Table 2:** Inter-observer agreement for UHRCT and CHRCT.

	Reader 1, 2		Reader 1, 3		Reader 2, 3	
	CHRCT	UHRCT	CHRCT	UHRCT	CHRCT	UHRCT
Lobulation sign	0.18	0.24	0.48	0.38	0.18	0.18
Spiculation	-0.01	-0.01	1.00	1.00	-0.01	-0.01
Pleural indentation	0.42	0.41	0.53	0.65	0.37	0.61
Bubble sign	-0.01	-0.01	-0.01	-0.01	0.66	1.00
Margin of nodule	0.38	0.51	0.27	0.51	0.42	0.46
Solid component	0.52	0.57	0.47	0.37	0.71	0.65
Diagnosis	0.39	0.42	0.34	0.52	0.25	0.45
Follow-up	0.22	0.27	0.39	0.42	0.21	0.32
Diagnostic Confidence	0.06	0.02	-0.02	0.17	-0.01	0.08

CHRCT, conventional high-resolution CT; UHRCT, ultra-high-resolution CT

### CT image of representative cases

**Fig 4:**
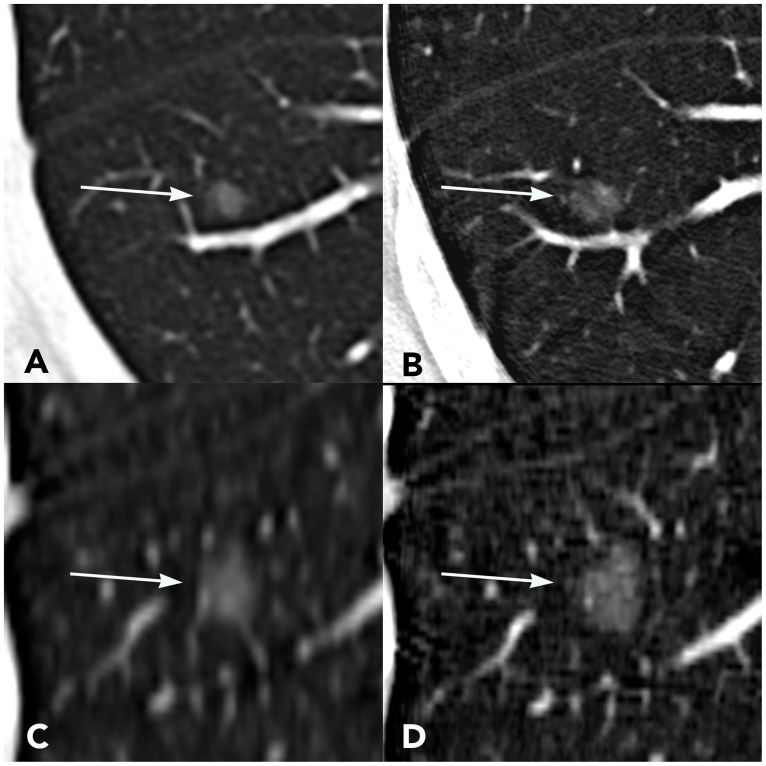
A 31-year-old male patient with AIS. The nodule is located in the dorsal segment of the right lower lobe of the lung. (A) Cross-sectional CHRCT image; (B) Cross-sectional UHRCT image; (C) Coronal CHRCT image; (D) Coronal UHRCT image.

**Fig 5:**
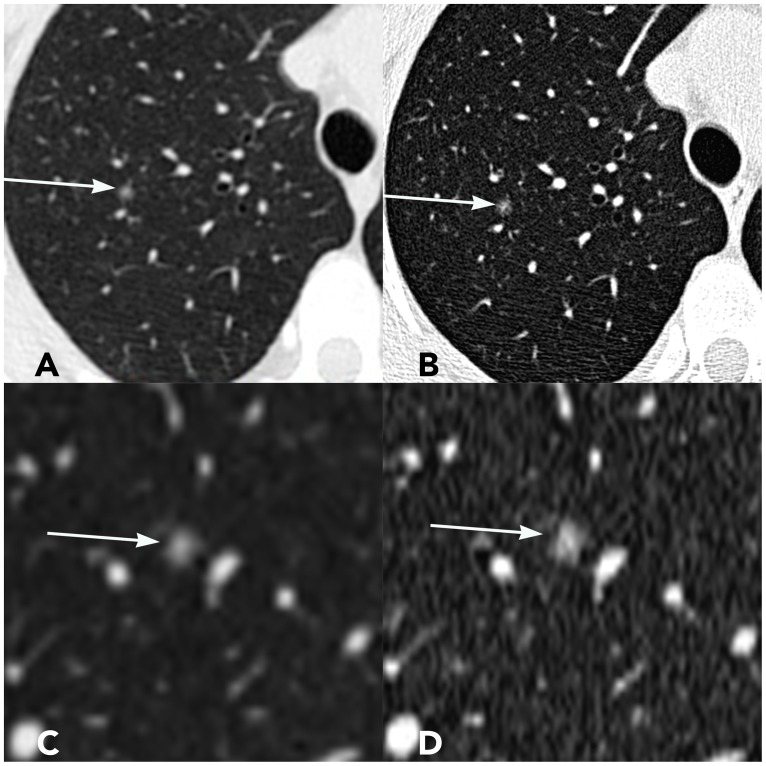
A 48-year-old female patient with AAH. The nodule (arrow) is located in the right lung apex. The maximan diameter is 3.60mm. (A) Cross-section CHRCT image (B) Cross-section UHRCT image (C) Sagittal CHRCT image (D) Sagittal UHRCT image.

Figs 4 and 5 show two representative cases. In cross sectional images and corresponding MPR images, the edge of nodules and solid internal components were shown more clearly on the UHRCT image than on the CHRCT images.

## Discussion

According to the US NCCN guidelines, resection is recommended for nodules larger than 8 mm in diameter, whereas follow-up is recommended for nodules less than 8 mm in diameter. Gierada et al. [11] reported that increase of the diameter threshold of positive nodules may reduce the false-positive rate of lung cancer screening. In this study, however, we found that the diameter of 62 nodules in 52 patients were primarily less than 8 mm with a mean maximum diameter of 6.18 ± 0.22 mm, which made it difficult to obtain consistent diagnoses among different doctors even after multiple follow-up sessions. In addition, indeterminate nodules may result in patients' extreme anxiety, frequent medical consultation, and seriously excessive examination. Therefore, it is important to provide a clear diagnosis so as to decrease the number of visits to the doctor as much as possible, regardless of the nodule's size.

In this study, a ultra-high-resolution CT is compared with CHRCT regarding image quality and analysis is based on whether it has an comparative advantage on the resulting diagnosis. To our knowledge, this prospective clinical study is the first evaluating the image quality and disagnosis accuracy for the detection of pulmonary nodules using UHRCT compared to that of CHRCT. Our results suggest that the UHRCT can provide significantly better image quality than CHRCT for margins of nodules and solid internal components. Other signs, such as the lobulation sign, spiculation, pleural indentation, and bubble sign are rarely detected due to the small size of the nodules. Therefore, further study should be performed to determine whether UHRCT has a higher sign detection rate or better image quality.

Nonsolid lung cancers may initially grow internally; and as the increase in the number of tumor cells, the nodules become more solid tumor appearances. It is reported that increase in soft tissue is caused by the tumor itself [12]. This indicates that internal growth of nonsolid nodules is crucial for the early diagnosis of lung cancer, and is also the primary cause of diagnosis inconsistency between physicians [13–15]. Edges of the nodules are used to distinguish between benign and malignant nodules: benign nodules often have smooth and sharp edges; infectious abnormality often have more blurred edges; and malignant pulmonary nodules usually have clear but not sharp edges. In this study, the edges of nodules and solid internal components were more clearly on the UHRCT images compared to that on the CHRCT images, which may help to improve the diagnoses of pulmonary nodules.

The diagnoses of two sets of the image made by the three radiologists in this study were not statistically different. The consistency in diagnosis may be due to most of the cases included in this study were pre-invasion lesions. Therefore, CT images contain many similarities, resulting in difficulty differentiating. With regard to the diagnostic confidence, three radiologists’ diagnostic confidence was significantly improved. Adequate diagnostic confidence will help to provide clear follow-up recommendations: 1) for patients diagnosed with malignancy early surgery should be recommended, regardless of the nodule size; and 2) for patients diagnosed as benign (e.g., granuloma) but will not eliminated within a short timeframe, patients should be clearly informed but follow-up is not necessarily recommended. This can reduce the psychological burden of patients. As expected, the results of this study showed that after UHRCT scanning, the number of cases requiring follow-up was significantly decreased, while the number of cases requiring surgery and no treatment was higher in comparison to those of CHRCT.

One of the possible reasons for the conflict results that there is no significant difference in the diagnosis but significantly differ in follow-up recommendations, may be related to the diagnostic confidence and the difficulty in diagnosis of early lung cancers. It is a challenge to differentiate AIS and AAH due to the similarity in CT images. Despite of the same diagnosis based on the two sets of images, the two sets of images may show a difference in density heterogeneity, which suggests a difference in number of cancer cells and degree of malignancy. Under this circumstance and taking into account the diagnostic confidence, radiologists may give different follow-up recommendations with the same diagnosis. For example, a nodule is diagnosed as AIS both on CHRCT images and UHRCT images. Due to UHRCT images can show a larger density heterogeneity, radiologists will be more inclined to diagnose the nodule as malignancy and have more confidence in diagnosis and make a decision to get surgery. Whereas on CHRCT images, radiologists will be more inclined to make more conservative judgments such as follow-up due to lacking confidence. Hence, we recommend UHRCT with small scan FOV scanning for undiagnosed nodules detected by screening.

The inter-observer agreement as assessed using the k test was different for each item. In these cases with the lobulation sign, spiculation, and bubble signs, the diagnostic confidence was poor. Firstly, it may be due to the difference in work experience among the radiologists [16, 17]. Moreover, it has been reported that even among experienced physicians, there is still substantial variability regarding the evaluations of pulmonary nodule [18]. This may be due to inherent interobserver variability is unavoidable for the diagnostic imaging. Secondly, the strong subjectivity of diagnostic confidence could be another reason for these differences. Finally, the number of nodules included in this study were relatively small and some remained undiagnosed even after several medical consultations with different doctors. Thus, there is a certain degree of difficulty in the diagnosis of such nodules.

The present study has several limitations. Firstly, the UHRCT scanner is generally more susceptible to the heartbeat, which means there may be more motion artifacts in UHRCT images. Furthermore, the noise of UHRCT of this study was higher. In this study, we used a higher tube current to reduce the image noise for the UHRCT scanner. Yasaka et al. [19] reported that HRCT with the new version of model-based iterative reconstruction and spatial resolution preference algorithm (MBIRn) can provide both the high spatial resolution and acceptable level of image noise. Further studies that combine UHRCT whth MBIRn should be conducted. Finally, the sample size of this study was relativley small and pathologic results were not obtained in all cases. Further studies with a larger number of samples are warranted.

### Conclusions

The findings suggest that UHRCT scanning with small scan FOV can provide a better image quality compared to CHRCT, particularly for subsolid nodules. In addition, UHRCT scanner can help to distinguish malignant pulmonary nodules from suspicious nodules during the follow-up periods and significantly reduce patient follow-up periods.
